# Enhancing the quality of surgical care through improved patient handover processes

**DOI:** 10.1186/s13037-025-00428-0

**Published:** 2025-03-14

**Authors:** Jessica M Ryan, Deborah A. McNamara

**Affiliations:** 1 RCSI StAR PhD Programme, School of Postgraduate Studies, 123 St. Stephen’s Green, Co, Dublin, Ireland; 2 The Bon Secours Hospital, Glasnevin Hill, Glasnevin, Co Dublin Ireland; 3 Office of the President, RCSI, 123 St. Stephen’s Green, Co Dublin, Ireland; 4 National Clinical Programme in Surgery, RCSI, 2 Proud’s Lane, Co Dublin, Ireland; 5 Department of Surgery, Beaumont Hospital, Beaumont, Co Dublin Ireland

**Keywords:** Handover, Continuity of care, Quality, Healthcare improvement, Implementation, Core outcome set

## Abstract

Surgical handover remains a high-risk process with no gold standard for practice despite 20 years of available guidance. Variability in practice is common, and poorly performed handover poses significant, yet avoidable, risk to patients. Research in this domain is underfunded with widely heterogenous methodology, meaning that the evidence base for better handover is deficient. In this correspondence, recommendations are made to address these shortcomings, including standardised operating procedures supported by electronic health records to enable staff training and audit. Prioritisation of the sickest patients at the handover outset and two-way, verbal communication, including a “read-back” to confirm that information is both transmitted and received. Rigorous evaluation of handover interventions before use, and discontinuation of practices that add no value. Lastly, a core outcome set for surgical handover is urgently needed to improve the comparability of studies. By clearly defining best practices and demonstrating the impact of interventions on patient outcomes, surgeons will be more inclined to adopt meaningful improvements in handover processes.

Surgical handover, or handoff, is the exchange of information between surgeons at the time of transfer of responsibility for a patient’s care [1] and is widely acknowledged to be hazardous due to challenges in safely transmitting complex information and context from one practitioner to the next [2]. Handovers have increased in frequency due to changing shift patterns and reductions in working hours among surgeons in training, yet practice has lagged in compensating for these changes. Surgical patients experience more handovers of care than any other patient group [2, 3] and evidence suggests that practice is highly variable [4, 5]. Handover-related issues with patient care are frequently occurring events [6] and staff report an unacceptable level of associated patient harm [7]. Poorly performed handover poses significant, yet avoidable, risk to patients.

In other safety critical industries, communication norms are standardised [8], taught, and often mandated by regulators [9]. In contrast, although handover guidance exists [10–14], high degrees of physician autonomy result in handover processes that are ill-defined, undocumented, and variable, even within the same organisation [1]. Communication errors are a key contributor to adverse outcomes in surgical patients, and when critical incidents are associated with communication failures, most are due to omissions of information [6, 15, 16]. Standardisation of communication in the immediate perioperative period by means of the safe surgery and other checklists has been well-evaluated and improved [17, 18], yet the literature on surgical handover remains strikingly heterogenous and of low quality [1]. The evidence base for better handover is deficient.

Although many healthcare staff perform handover, surgery differs from other disciplines due to time pressures, urgency of care, the rapid turnover of patients, and the multiplicity of hospital locations where care is delivered. As a result, process improvement in surgery is difficult and can be hazardous as uncontrolled change may paradoxically reduce safety by alienating staff and eliminating work-arounds that increase safety [19]. At an organisational level, the requirement for scarce healthcare staff to devote time to handover has clear cost implications for employers. Despite the adverse consequences of error, there is little evidence that support for higher quality handover is prioritised [7]. An appropriate balance must be struck between inadequate, abbreviated transfers of care, and prolonged handover meetings leading to delays in care delivery and staff overtime.

Surgeons are likely to support change if it demonstrably improves patient outcomes [20]. Considering the importance of non-technical skills in surgery [21–23], this support would likely encompass both technical and communication-focused interventions. Yet despite the intuitive assumption that better handovers yield better patient outcomes, establishing this link remains challenging, and research in this domain has historically been underfunded [1]. 

So, how do we move forward to improve surgical handover? Firstly, at a minimum, hospitals must establish standard operating procedures for handover [10, 14] that are required practice for all surgical staff and supported by an electronic healthcare record [10, 11, 13]. This should enable high quality training of staff, simulation, audit, improved efficiency and reduced error with process automation [24, 25], and reduction of unnecessary documentation.

Every surgical handover must clearly identify the sickest patients and the highest priorities for the incoming team in a highly reliable way [10–14]. This not only has the potential to improve patient outcomes [1], but may increase learning opportunities at handover for surgeons-in-training by making higher order thinking more explicit.

Surgical handover must involve verbal, two-way communication [12–14, 26], it is not just a document. Systematic inclusion of a “read-back” in handover communications ensures the receiver has understood the information [27]. The aphorism “*the single biggest problem with communication is the illusion that it has taken place*”, attributed to Irish playwright George Bernard Shaw, summarises a key challenge in communication-based interventions caused by egocentric processes [28]. Safe care means that the surgeon handing over is certain that information has not just been transmitted, but also received.

Modifications to surgical handover processes need to be properly supported [29]. Not all change leads to improvement [30], and communication-based interventions in surgery must be exposed to the same rigorous analysis as technical innovations. The burden of untested new practices cannot be placed upon surgical shoulders at a time when administrative workloads have never been higher. Introducing changes to surgical handover processes requires time, perhaps our scarcest resource, and is only worthwhile when there is clear evidence of patient benefit. Improved practices should involve rigorous analysis of the costs and benefits of change, but also a willingness to remove work that adds no value to patient care, known as de-innovation [31]. 

Finally, a core outcome set for handover research is needed. A recent review reported over 50 outcomes used to evaluate the effectiveness of surgical handover interventions [1]. This heterogeneity makes it difficult, if not impossible, to directly compare study results and advance the development of a gold standard for surgical handover, which does not currently exist [32]. There remains little clarity about which components of handover, and which handover outcomes [33], are critical, and a gap in our understanding of the impacts of specific handover practices on patients. Changing handover practice is complex, but when best practice is clearly defined and the impact on patient outcomes can be demonstrated, surgeons will change their practice.

## Data Availability

No datasets were generated or analysed during the current study.
